# Vaginal Laser Treatment on Stress Urinary Incontinence: A Randomized Controlled Trial

**DOI:** 10.1007/s00192-025-06160-8

**Published:** 2025-05-23

**Authors:** Lok Wun Yim, Osanna Yee Ki Wan, Daniel Wong, Man Sum Tam, Yun Ting Lee, Kai Wan Lee, Kwong Wai Choy, Symphorosa Shing Chee Chan, Rachel Yau Kar Cheung

**Affiliations:** 1 Department of Obstetrics and Gynaecology, Faculty of Medicine, The Chinese University of Hong Kong, 1E, Prince of Wales Hospital, Ma Liu Shui, Hong Kong; 2 Department of Obstetrics and Gynaecology, Pamela Youde Nethersole Eastern Hospital, Chai Wan, Hong Kong; 3 Department of Obstetrics and Gynaecology, Princess Margaret Hospital, Lai Chi Kok, Hong Kong

**Keywords:** Er:YAG, Randomized controlled trial, Stress urinary incontinence, Vaginal laser

## Abstract

**Introduction and Hypothesis:**

This study evaluates the efficacy of vaginal Erbium:YAG laser treatment for stress urinary incontinence (SUI) compared with sham treatment over 12 months.

**Methods:**

This single-blinded, multicenter randomized controlled trial involved women diagnosed with urodynamic SUI at three urogynecology centers in Hong Kong. Participants were randomly assigned to receive either vaginal laser therapy (two sessions, 4 weeks apart) or sham treatment. The primary outcome was the reduction in Pelvic Floor Distress Inventory (PFDI) Urinary Distress Inventory (UDI) Stress subscale scores at 6 and 12 months. Secondary outcomes included urinary incontinence episodes, severity from bladder diaries, visual analog scale (VAS) scores, and scores from the Pelvic Floor Impact Questionnaire Urinary Impact Questionnaire and the International Consultation on Incontinence Questionnaire Urinary Incontinence Short Form.

**Results:**

Out of 114 screened women, 75 were randomized. Significant reductions in PFDI UDI stress subscale scores were shown in the treatment group at the 6-month and 12-month follow-up (*p* < 0.001 at 6 months; *p* < 0.001 at 12 months) but was not significant in the control group. Significant reductions were observed in the treatment group at the 6-month and 12-month follow-up in all secondary outcome parameters whereas the Pelvic Floor Impact Questionnaire UDI score and the VAS score of SUI severity at both 6 months and 12 months were not significantly reduced in the control group. There was no significant difference in both primary or secondary outcomes between groups.

**Conclusions:**

Vaginal Erbium: YAG laser improved SUI symptoms and shows potential as a minimally invasive option, but lacks significant differences from sham treatment, necessitating further research.

## Introduction

Urinary incontinence (UI) is a prevalent health care concern. A systematic analysis estimated that UI affects 25–45% of the female population globally, with the highest prevalence observed among older women in Asia [1]. The prevalence of UI in women in our locality was reported to be as high as 66% [2]. The most common type of UI is stress urinary incontinence (SUI). Although it is not life threatening, UI significantly diminishes health-related quality of life and imposes a considerable financial burden on women and health care systems [3].

Current nonsurgical treatment options for SUI include physical therapy, dietary modifications, and the use of pessaries [4]. Among these, pelvic floor exercise (PFE) is the most recommended conservative approach owing to its positive outcomes, minimal side effects, lower hospital costs, and the fact that it reduces UI episodes by 70% in affected women [5]. PFE has demonstrated superiority over no active intervention for treating UI [6]; however, the success of conservative management largely depends on women’s compliance.

The advantages of surgical treatment for SUI are supported by numerous studies [7]. However, potential complications associated with surgery, such as bleeding, infection, bladder or urethral injury, pain, voiding dysfunction, and particularly mesh-related risks [8], may cause many women to hesitate before seeking alternative options, highlighting the need for safer, innovative nonsurgical treatments for SUI.

Vaginal laser therapy has emerged as a potential alternative in recent decades. Initially, it was used to alleviate symptoms of the genitourinary syndrome of menopause [9], but it has since gained attention as a treatment for SUI, overactive bladder (OAB), and pelvic organ prolapse (POP) [10].

There are two main types of vaginal lasers: non-ablative erbium-doped yttrium aluminum garnet (Erbium:YAG) lasers and ablative carbon dioxide (CO_2_) lasers [11]. Both types are aimed at achieving collagen remodeling in the subepithelial connective tissue, albeit through different mechanisms [12]. The CO_2_ laser, operating at 10,600 nm, causes tissue denaturation, leading to collagen and elastin fiber remodeling [13]. In contrast, the Erbium:YAG laser also operates at 10,600 nm but has a significantly higher water absorption capacity—approximately 10 to 15 times greater than that of the CO_2_ laser. This property allows it to create a more profound secondary thermal effect, facilitating controlled heating of the targeted mucous membrane in the vaginal wall while preserving the vaginal epithelium, thus promoting collagen fiber contraction and remodeling [12]. Histological studies provide empirical evidence supporting collagen remodeling resulting from both laser therapies [14].

Numerous studies have explored the effectiveness of CO_2_ laser therapy for managing SUI symptoms. However, these studies often suffer from small sample sizes, varied designs, different follow-up durations, and inconsistent outcomes [14–17]. Previous trials involving vaginal erbium laser (VEL) have yielded promising results, with a favorable risk–benefit profile [18]. A recent European sham-controlled clinical trial concluded that VEL significantly improves SUI symptoms compared with sham treatment, suggesting that it should be considered a nonsurgical option for women with SUI [19]. Nevertheless, in Asian population, there is a pressing need for high-quality studies on VEL for women with SUI.

We aimed to compare the effects of vaginal Erbium:YAG laser treatment against sham treatment over a longer follow-up period in a multicenter setting in Asia, involving participants with urodynamic SUI.

## Materials and Methods

This was a single-blinded multicentered randomized controlled trial (RCT), with women recruited in three urogynecology centers in Hong Kong from December 2018 to May 2020. All women aged over 18 years presenting to the urogynecology clinic for UI symptoms were invited. Women underwent a urodynamic study of uroflowmetry and cystometry. Only women diagnosed with urodynamic stress incontinence (USI) were recruited. Women who had mixed UI with predominant urgency urinary incontinence, recurrent USI in which previous surgical treatment had failed, e.g., tension-free vaginal tape surgery or injection of bulking agent, pregnant or lactating women, women with stage II or above POP, women with undiagnosed vaginal bleeding, and women currently using photosensitive drugs were excluded. Written informed consent was obtained and the privacy rights of the study participants were observed.

Information on demographic and urinary symptoms was obtained before any intervention. The attending gynecologist assessed women for any concomitant POP using the Pelvic Organ Prolapse Quantification (POPQ) system, strength of pelvic floor muscle contractions (PFMCs) using a grading scale from 0 (no contraction) to 5 (strong contraction); and anal tone on a scale of 0 to 5. Urodynamic studies were conducted according to the international guidelines [20]. Women were asked to complete the validated Chinese Pelvic Floor Distress Inventory (PFDI), the Pelvic Floor Impact Questionnaire (PFIQ) [21], the International Consultation on Incontinence Questionnaire (ICIQ), the Female Sexual Function Index (FSFI), and the Short Form Health Survey (SF-12). They were also asked to perform a 3-day bladder diary.

Randomization was performed in a 2:1 ratio of treatment group: control group, with a block size of 3, by computer-generated random number series in sealed envelopes. None of the personnel involved in the study had any influence on the randomization procedure and all investigators who performed pre- and post-treatment assessment were blinded to the allocation sequence. Women included in the study were masked with the use of a sham procedure. Owing to the nature of the study, investigators who performed the vaginal laser therapy or sham procedure could not be masked.

Recruited women were randomized into two groups following eligibility assessment. The treatment group underwent vaginal laser therapy consisting of two sessions spaced 4 weeks apart, with each session lasting 20 min. Treatments were administered using an Er:YAG infrared laser system (Juliet, Jena, Germany) equipped with a Steri-Spot handpiece featuring a 90° reflecting gold-coated mirror. Each treatment session was performed in two distinct phases. The first phase employed an ablative fractional approach using GYN C Mode, which delivered a fluence of 5–35 J/cm^2^ with 300-µs pulse durations at intervals of 0.5–2 s. During this phase, the handpiece was inserted into the cervix/vaginal vault (7–10 cm depth) with the mirror positioned upward, then systematically rotated 45° (line-to-line alignment) between laser pulses until completing a full 360° rotation (eight pulses), after which it was withdrawn 1 cm. This sequence was repeated until the handpiece's final centimeter marker became visible outside the vaginal opening.

The second phase utilized thermal treatment through GYN W Mode, applying 6–12 J/cm^2^ fluence with longer 1000-µs pulses at the same 0.5- to 2-s intervals. The handpiece was reinserted to the original depth, and treatment proceeded with finer 22.5° rotations (line-to-dot alignment) between pulses. After completing each 360° rotation (requiring 16 pulses), the handpiece was withdrawn 0.5 cm, repeating the process until the marker became externally visible. Meanwhile, the control group received a sham procedure designed to mimic the treatment experience without therapeutic effects. Using identical session timing and frequency, the sham procedure employed zero-intensity settings while playing recorded laser system sounds through an amplifier placed on the device to maintain participant blinding throughout the study.

Follow-up visits with comprehensive assessments were scheduled at 6 weeks, 3 months, 6 months, and 12 months post-intervention. During each visit, women were requested to bring back their bladder and PFE diaries, along with a symptoms calendar. They were asked to complete the PFDI, PFIQ, ICIQ, FSFI, and SF-12. The tolerability of treatment was assessed through subjective pain scores (ranging from 1 to 10) and any complications or adverse effects were evaluated after each laser session. Women also provided subjective evaluations of their treatment outcomes, with options ranging from"much better"to"much worse,” and the severity of SUI measured by a 10-cm Visual Analog Scale (VAS) at each visit. Clinical assessments at each visit involved the evaluation of PFMCs and anal tone. These multifaceted evaluations were aimed at providing a thorough understanding of treatment efficacy and women-reported outcomes over the designated follow-up periods.

The primary outcome was the difference in reduction in the PFDI Urinary Distress Inventory (UDI) Stress subscale scores between groups at the 6-month and 12-month follow-ups. Secondary outcomes comprised the median number of SUI episodes over 24 h based on a 3-day bladder diary, the severity of SUI symptoms measured by a 10-cm VAS, subjective improvement in SUI symptoms, reductions in urinary impact questionnaire (UIQ) scores of the PFIQ, and changes in other PFDI and PFIQ domains. Any subsequent medical or surgical treatments were collected at the 12-month follow-up visit.

### Ethics Approval and Clinical Trial Registration

The study was carried out in accordance with the principles of the Declaration of Helsinki. Ethics approval number was CREC 2018.387-T. This RCT was registered at the Centre for Clinical Research and Biostatistics (Chi-CTR-1900021044) and the results were reported according to the Consolidated Standards of Reporting Trials statement.

### Sample Size Calculation

From our previous local study, the baseline PFDI UDI stress subscale of women diagnosed with USI was about 36 (SD 23) [22]. The minimal important difference of women who received continent surgery was reduced by one SD, which was −20. Assuming that the reduction of the PFDI UDI stress subscale score at 12 months was 20 and 5 for the treatment group and control group respectively, the difference in the scores between the two groups would be 15. With alpha of 0.05 and a power of 80%, and the treatment to control group ratio of 2 to 1, the estimated sample size was 42 for the treatment group and 21 for the control group. Assuming a drop-out rate of 15%, 50 women were needed for the treatment group and 25 women for the control group.

### Statistical Analysis

Data were analyzed primarily according to the intention-to-treat (ITT) principle. Missing data were handled using the last observation carried forward (LOCF) method. The LOCF is a commonly used approach for imputing data in the presence of dropouts. Descriptive analysis was conducted to analyze the demographic data, as well as the primary and secondary outcomes. For the indices of the primary and secondary outcomes, the Wilcoxon signed-rank test was used to compare the changes in indices at 6 months and 12 months with baseline within the same group. The Mann–Whitney *U* test was used to compare the indices between the laser and sham groups. The Chi-squared test or Fisher’s exact test was used for categorical data. Linear logistic regression analysis was applied, if appropriate, to assess the impact of various factors, including age, parity, BMI, PFDI UDI, and PFIQ UIQ scores, on subjective improvement. The significance level was set at 0.05.

## Results

**Fig. 1:**
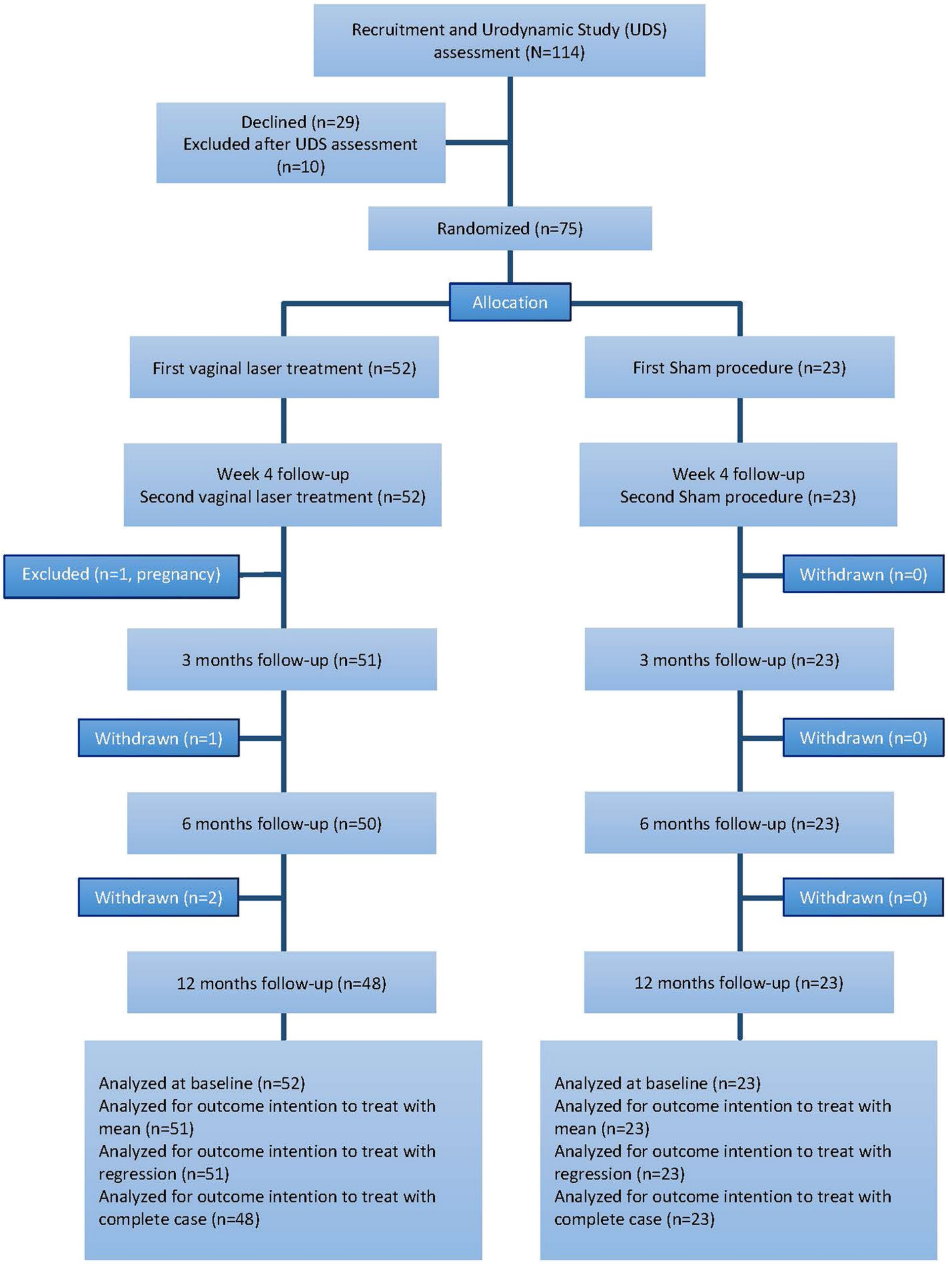
The trial profile of randomized women in the laser treatment group and the sham control group

A total of 114 women were invited for studies from December 2018 to May 2020. Seventy-five eligible women were randomized, with 52 in the laser treatment group and 23 in the sham control group. All women completed the two treatment sessions, except for one who was excluded from the study after completion of vaginal laser treatment because she was found to be pregnant at the 3-month follow-up. The overall loss to follow-up rate was 4% (*n* = 3). There was no deviation from the protocol (Fig. 1). Baseline demographics and characteristics were similar in the recruited and excluded women, as well as between the treatment group and the control group, including baseline PFDI UDI score and PFIQ UIQ score (Table 1).

**Table 1:** Demographics and baseline characteristics

	All participants (*N* = 75)	Laser group (*n* = 52)	Sham group (*n* = 23)	*p* value
Age at recruitment (years)	54.8 ± 8.9	54.6 ± 8.7	55.4 ± 9.6	0.734
Body mass index (kg/m^2^)	26.4 ± 4.1	26.8 ± 4.4	25.4 ± 3.0	0.283
Postmenopausal	50 (66.7)	34 (65.4)	16 (69.6)	0.929
Pelvic floor muscle contraction	4.0 ± 0.8	4.0 ± 0.8	3.9 ± 0.8	0.725
PFDI UDI stress subscale	37.5 (29.2–54.2)	41.7 (29.2–54.2)	37.5 (33.3–62.5)	0.716
PFDI UDI score	83.2 (53.3–110.8)	79.9 (52.1–107.5)	84.9 (55.8–124.9)	0.199
PFIQ UIQ score	79.4 (47.0–171.3)	75.8 (33.3–154.2)	97.6 (60.0–191.0)	0.123
SUI VAS score	6.4 (4.8–7.8)	6 (3.7–7.2)	7.2 (5–8.95)	0.082
Number of SUI in 24 h in 3-day bladder diary	1 (0.7–1.3)	1 (0.7–1.3)	1 (0.6–1.7)	0.647
Total daily fluid intake (ml)	1745 ± 583	1746 ± 610	1744 ± 527	0.992
Total daily voiding volume (ml)	1947 ± 616	1985 ± 645	1859 ± 549	0.428
Number of daytime voids	6.6 ± 2.2	6.6 ± 2.4	6.6 ± 1.6	0.768
Number of nocturia episodes	0.8 ± 0.8	0.7 ± 0.8	0.9 ± 0.8	0.440
Maximum void volume (ml)	398 ± 133	410 ± 129	370 ± 141	0.247
Pelvic floor exercise (sets/day)	2.0 ± 1.7	1.8 ± 1.4	2.5 ± 2.0	0.097
Anal tone	4.8 ± 0.5	4.8 ± 0.5	4.7 ± 0.6	0.199
Subjective severity				
Mild	64 (85.3)	46 (88.5)	18 (78.3)	0.085
Moderate	7 (9.3)	5 (9.6)	2 (8.7)	
Severe	2 (6.7)	0 (0)	2 (8.7)	

*PFDI* Pelvic Floor Distress Inventory, *PFIQ* Pelvic Floor Impact Questionnaire, *UDI* Urinary Distress Inventory, *UIQ* Urinary Impact Questionnaire, *ICIQ-UI SF* International Consultation on Incontinence Questionnaire Urinary Incontinence Short Form, *VAS* Visual Analog Score

Data in median (interquartile range) or mean ± SD

*p* values refer to comparison of median scores or mean between groups by Mann–Whitney *U* test

### Primary Outcome

The PFDI UDI Stress subscale scores decreased in the treatment group from 4 weeks to 3 months and were significantly reduced at the 6-month and 12-month follow-ups. Among those with complete data, the PFDI UDI Stress subscale score reduced from 41.7 (interquartile range [IQR] 29.2–54.2) at baseline to 33.3 (IQR 20.8–41.7) at 6 months (*p* < 0.001) and from 41.7 (IQR 29.2–54.2) at baseline to 27.1 (IQR 16.7–45.8) at 12 months (*p* < 0.001). In the ITT analysis using LOCF, the PFDI UDI Stress subscale score changed from 41.7 (IQR 29.2–54.2) to 33.3 (IQR 20.8–41.7) at 6 months (*p* < 0.001) and from 41.7 (IQR 29.2–54.2) to 25.0 (IQR 16.7–45.8) at 12 months. In contrast, the control group showed similar stress subscale scores that remained unchanged at 6 and 12 months (Table 2).

**Table 2:** Primary and secondary outcome of women in both the laser treatment group and the sham control group

		Baseline	4 weeks	3 months	6 months	*p* value	12 months	*p* value**
Primary outcome								
PFDI UDI Stress subscale score								
ITT by LOCF	Laser (*n* = 51)	41.7 (29.2–54.2)	33.3 (25.0–45.8)	33.3 (16.7–41.7)	33.3 (20.8–41.7)	< 0.001	25.0 (16.7–45.8)	< 0.001
	Sham (*n* = 23)	37.5 (33.3–62.5)	37.5 (29.2–54.2)	41.6 (20.8–58.3)	37.5 (19.8–58.3)	0.24	37.5 (25.0–54.2)	0.17
	p*				0.21		0.08	
Completed case	Laser (*n* = 48)	41.7 (29.2–54.2)	33.3 (25.0–45.8)	33.3 (16.7–42.7)	33.3 (20.8–41.7)	< 0.001	27.1 (16.7–45.8)	< 0.001
	Sham (*n* = 23)	37.5 (33.3–62.5)	37.5 (29.2–54.2)	39.6 (19.8–58.3)	37.5 (19.8–58.3)	0.24	37.5 (25.0–54.2)	0.17
	p*				0.21		0.14	
Secondary outcome								
PFDI UDI Score								
ITT by LOCF	Laser (*n* = 51)	79.9 (52.1–107.5)	60.9 (45.8–87.4)	64.6 (40.8–105.8)	60.4 (35.6–102.9)	< 0.001	52.5 (31.7–90.2)	< 0.001
	Sham (*n* = 23)	84.9 (55.8–124.9)	80.7 (47.5–129.9)	74.9 (46.8–131.5)	87.3 (29.7–116.7)	0.10	60.4 (34.2–121.9)	0.09
	p*				0.19		0.10	
Completed case	Laser (*n* = 48)	79.9 (52.1–107.5)	60.9 (45.8–87.4)	56.6 (36.6–101.0)	56.2 (40.7–95.4)	< 0.001	51.2 (30.9–86.4)	< 0.001
	Sham (*n* = 23)	84.9 (55.8–124.9)	80.7 (47.5–129.9)	74.9 (46.8–131.5)	87.3 (29.7–116.7)	0.10	60.4 (34.2–121.9)	0.09
	p*				0.30		0.13	
PFIQ_UIQ score								
ITT by LOCF	Laser (*n* = 51)	75.8 (33.3–154.2)	68.8 (32.2–105.7)	62.0 (26.0–106.8)	64.4 (24.1–99.0)	0.05	46.6 (16.0–89.9)	< 0.001
	Sham (*n* = 23)	97.6 (60.0–191.0)	74.4 (32.4–167.7)	65.0 (22.8–122.9)	89.7 (38.2–141.3)	0.011	51.7 (20.4–161.9)	0.022
	p*				0.24		0.36	
Completed case	Laser (*n* = 48)	75.8 (33.3–154.2)	68.8 (32.2–105.7)	62.1 (30.0–114.6)	64.4 (25.5–100.3)	0.012	49.4 (16.0–96.7)	< 0.001
	Sham (*n* = 23)	97.6 (60.0–191.0)	74.4 (32.4–167.7)	65.0 (22.8–122.9)	89.7 (38.2–141.3)	0.011	51.7 (20.4–161.9)	0.022
	p*				0.22		0.46	
ICIQ-UI SF score								
ITT by LOCF	Laser (*n* = 51)	12.5 (9–14)	10 (7–13)	9 (7–14)	9 (7.25–12)	< 0.001	9 (6–13)	< 0.001
	Sham (*n* = 23)	14 (11–15)	12 (9–14)	10.5 (6.3–14)	10.5 (6.8–14.25)	0.018	10.5 (7.8–14.3)	0.01
	p*				0.32		0.21	
Completed case	Laser (*n* = 48)	12.5 (9–14)	10 (7–13)	9 (7–14)	9 (7–12)	0.01	9 (6.25–13)	< 0.001
	Sham (*n* = 23)	14 (11–15)	12 (9–14)	10.5 (6.3–14)	10.5 (6.8–14.3)	0.018	10.5 (7.8–14.3)	0.01
	p*				0.32		0.16	
VAS score of severity of SUI								
ITT by LOCF	Laser (*n* = 51)	6 (3.7–7.2)	5.1 (2.7–7.5)	4.7 (2.0–6.8)	4.7 (2.8–5.8)	0.004	5 (3.3–6.6)	0.041
	Sham (*n* = 23)	7.2 (5–9.0)	4.75 (3–7.4)	5 (2.0–8.5)	4.8 (2.5–9.2)	0.22	5 (3.3–7.5)	0.005
Completed case	Laser (*n* = 48)	6 (3.7–7.2)	5.1 (2.7–7.5)	4.7 (2.2–6.7)	4.65 (2.9–5.8)	0.005	5 (2.8–6.7)	0.042
	Sham (*n* = 23)	7.2 (5–9.0)	4.75 (3–7.4)	5 (2–8.5)	4.8 (2.5–9.2)	0.22	5 (3.3–7.5)	0.005
Episodes of SUI								
ITT by LOCF	Laser (*n* = 51)	1 (0.7–1.3)	0.8 (0.3–1.0)	1 (0.3–1.3)	0.7 (0–1.1)	0.016	0.67 (0–1.0)	0.002
	Sham (*n* = 23)	1 (0.6–1.7)	1 (0.6–1.8)	0.5 (0–1.0)	0.7 (0–1.5)	0.034	0.3 (0.3–1.0)	0.014
Completed case	Laser (*n* = 48)	1 (0. 7–1.3)	0.8 (0.3–1.0)	1.0 (0.3–1.3)	0.7 (0–1.0)	0.006	0.7 (0–1.0)	0.003
	Sham (*n* = 23)	1 (0.6–1.7)	1 (0.6–1.8)	0.5 (0–1.0)	0.7 (0–1.5)	0.034	0.3 (0.3–1.0)	0.014

Data are median (interquartile range)

Missing data generated by last observation carried forward method in the ITT analysis

There is no significant difference in any of the median scores between the laser group and the control group by Mann–Whitney *U* test (*p* > 0.05)

*PFDI* Pelvic Floor Distress Inventory, *PFIQ* Pelvic Floor Impact Questionnaire, *n* Urinary Distress Inventory, *UIQ* Urinary Impact Questionnaire, *ICIQ-UI SF* International Consultation on Incontinence Questionnaire – Urinary Incontinence Short Form, *VAS* Visual Analogue Scale, (*SUI* stress urinary incontinence, *ITT* intention-to-treat

*p values refer to comparison of scores between laser and sham groups

**p values refer to comparison of scores with the baseline scores within groups by Wilcoxon signed-rank test

### Secondary Outcome

The secondary outcomes were listed in Table 2. The PFDI UDI score in the treatment group decreased from 4 weeks to 3 months and was significantly reduced at the 6-month and 12-month follow-ups. In those with complete data, the score decreased from 79.9 (IQR 52.1–107.5) at baseline to 56.2 (IQR 40.7–95.4) at 6 months (*p* < 0.001) and to 51.2 (IQR 30.9–86.4) at 12 months (*p* < 0.001). In the ITT analysis with LOCF, scores reduced from 60.4 (IQR 35.6–102.9) at 6 months (*p* < 0.001) and to 52.5 (IQR 31.7–90.2) at 12 months (*p* < 0.001). The control group exhibited similar scores that remained unchanged at both follow-ups.

The PFIQ-UIQ scores and ICIQ-UI SF scores in the treatment group decreased from 4 weeks to 3 months and were significantly reduced at the 6-month and 12-month follow-ups. Significant reductions were also observed in the control group, in both complete data and ITT analyses. Overall, 60% of our patients reported that they were sexually inactive and did not provide the data on FSFI score. Therefore, FSFI scores were not reported. The severity of SUI, assessed by VAS, decreased in the treatment group from 4 weeks to 3 months and was significantly reduced at the 6-month and 12-month follow-ups. In the control group, the VAS remained similar at 6 months, there was no significant reduction but increment was observed instead at 12 months. The median number of episodes of SUI in 24 h, as recorded in a 3-day bladder diary, decreased in the treatment group from 4 weeks to 3 months and was significantly reduced at the 6-month and 12-month follow-ups.

There is no significant difference in all PFDI, PFIQ, and ICIQ scores between the treatment group and the control group by Mann–Whitney *U* test (*p* > 0.05). The possible confounding factors including age, menopausal status, BMI, and pelvic floor exercise compliance were input for logistic regression with no significant association identified.

Subgroup analysis was performed according to the severity of SUI symptoms. Women with mild to moderate symptoms (with a baseline VAS score of less than 8 or a total PFDI UDI score between 1 and 100) were identified. There was no significant difference in the PFDI UDI stress subscale, PFIQ-UIQ, and ICIQ-UI SF between the treatment group and the control group at all follow-up visits.

Subjective improvement of SUI symptoms reported by both groups of women were listed in Table 3. There was no significant difference in the subjective improvement of SUI symptoms between the treatment group and the sham group at 4 weeks (*p* = 0.08), 3 months (*p* = 0.34), 6 months (*p* = 0.99), and 12 months (*p* = 0.46) post-treatment.

**Table 3:** Subjective improvement in stress urinary incontinence symptoms

	At 4 weeks’ follow-up			At 3 months’ follow-up			At 6 months’ follow-up			At 12 months’ follow-up		
	Mild/much improved	Not improved	*p* value	Mild/much improved	Not improved	*p* value	Mild/much improved	Not improved	*p* value	Mild/much improved	Not improved	*p* value
Laser (*n* = 47)	27 (57.4%)	20 (42.6%)	0.075	25 (53.2%)	22 (46.8%)	0.34	25 (53.2%)	22 (46.8%)	0.99	21 (44.7%)	26 (55.3%)	0.46
Sham (*n* = 23)	8 (34.8%)	15 (65.2%)		15 (65.2%)	8 (34.8%)		12 (52.2%)	11 (47.8%)		11 (47.8%)	12 (52.2%)	

*p* value, comparison of outcomes with overall improvement and no improvement between vaginal laser and control groups by Chi-squared test

At the 12-month follow-up, 44% of women (21 women in the treatment group and 10 women in the control group) opted to continue pelvic floor exercise alone, with the remaining 55% (26 women in the treatment group, 13 women in the control group) were planned for continence surgery. No statistical difference was found between the two groups (*p* = 0.60).

### Adverse Effects

The pain scores during the procedure and immediately after the procedure were significantly higher in the treatment group than in the control group at the first session (during the procedure: pain score 5.24 [SD 2.46] versus 0.71 [SD 1.59]; *p* < 0.01; after the procedure: 1.68 [SD 2.02] versus 0.37 [SD 0.90]; *p* < 0.01 respectively), and also at the second session of treatment (during the procedure: pain score 5.55 [SD 2.58] versus 0.85 [SD 2.01]; *p* < 0.01; after the procedure: 2.13 [SD 2.37] versus 0.46 [SD 1.47]; *p* < 0.01 respectively). There were more women complaining of vaginal spotting after vaginal laser treatment: 30 women (59%) in the treatment group versus 3 (13%) in the control group (*p* < 0.001); 29 (57%) in the treatment group versus 1 (4%) in the control group (*p* < 0.001) after the first and second sessions respectively. No major adverse events were reported otherwise.

## Discussion

The main finding of this multicenter, randomized controlled trial is that vaginal erbium laser treatment (two treatment sessions 4 weeks apart) resulted in significant improvements in SUI outcomes in the treatment group after 6 months, lasting up to 12 months post-treatment. However, a clinically relevant superior effect of the treatment over the sham was not demonstrated. Subjective measures included validated questionnaires and the VAS, whereas the objective measure was the number of SUI episodes over 24 h from the 3-day bladder diary. Our primary results confirm that vaginal erbium laser treatment is an effective option for SUI, aligning with previously published data on similar treatments [23–25], but the improvement was not significantly different when compared with the sham group. No serious adverse effects were recorded.

To our knowledge, this is the first sham-controlled randomized clinical trial of vaginal erbium laser treatment for SUI in an Asian population, featuring a long follow-up of up to 12 months after the primary intervention. Previous studies have indicated that treatment effects are dose responsive, suggesting that participants might benefit from additional treatment sessions [26]. According to available data, multiple treatment sessions (more than two) can result in sustained effects lasting 1–2 years [25]. A previous study confirmed the durability of the effect on SUI symptoms in the active group at the 12-month follow-up, after two vaginal erbium laser sessions 1 month apart [19]. Our study further substantiates the lasting impact of vaginal erbium laser treatment for SUI up to 12 months, as demonstrated by improvements in the PFDI UDI stress subscale, PFDI UDI total score, PFIQ UIQ, ICIQ-UI SF, VAS, and the number of SUI episodes.

Some improvements were also noted in the control group, including significant reductions in PFIQ UIQ, ICIQ-UI SF scores, VAS, and urinary frequency. These changes may be attributed to the effect of practicing pelvic floor exercise and a placebo effect of this magnitude, which was anticipated based on prior studies [17] and accounted for in the sample size calculations for this trial. Understanding the magnitude of the placebo effect is not only valuable for designing future clinical trials but also provides context for interpreting results from uncontrolled or active-controlled studies [27].

In a short-term, single-center study, the effect of vaginal erbium laser treatment was comparable with sham treatment [24]. However, this study involved only one treatment session and followed participants for 3 months, using the ICIQ-UI SF questionnaire as the primary outcome measure. The ICIQ-UI SF scores significantly improved in the treatment group compared with the sham control group. A multicenter sham-controlled trial involving women with USI employed two vaginal erbium laser sessions spaced 1 month apart [19], with a follow-up period of 6 months. This study found a significantly higher treatment success rate, defined as a greater than 50% reduction in pad weight from baseline, in the treatment group. However, no significant differences were observed in the median ICIQ-UI SF scores between the laser and control groups at 6 months. In our study, there was no significant difference in median scores for the PFDI-UDI stress subscale, PFDI-UDI scores, PFIQ-UIQ, ICIQ-UI SF, VAS, and daily frequency of SUI between the laser and control groups at 6 and 12 months. Despite the objective outcomes (e.g., reduction in pad weight) being observed at 6 months, the effect may not have been substantial enough to influence women's subjective symptoms, as evidenced by validated questionnaires.

Previous research indicated that women experiencing mild to moderate SUI are good candidates for vaginal laser therapy. A subgroup analysis focusing on baseline SUI severity in women receiving vaginal Erbium:YAG laser treatment was conducted by Kuszka et al. [25]. The study included women diagnosed with either SUI or mixed UI with a predominance of SUI. SUI severity was assessed using Stamey’s incontinence scoring system [28]. Following two laser sessions, the ICIQ-UI SF scores showed significant improvement in women with mild and moderate symptoms but not those with severe symptoms. In our study, women with mild to moderate symptoms did not show a significant difference in their improvement between two groups. This inconsistency may be attributed to the fact that, although our sample size was adequate based on initial calculations, it may lack sufficient power for subgroup analysis. Additionally, women with mixed UI were excluded from our study, which may also account for the different findings.

At the 12-month follow-up, the PFDI UDI demonstrated improvement in SUI symptoms among the treatment group; however, there was no statistically significant difference in the plan of further treatment with continence surgery between the two groups. We understand that several factors might affect women’s choice of treatment, including comorbidity, individual symptoms such as the severity of SUI, the presence of OAB symptoms, and previous treatment response [29].

Laser treatment for SUI has been found to be safe in our study and others [15, 18], with no reported serious side effects even with a long-term follow-up of 12–36 months. Minor immediate adverse effects, such as a brief stinging sensation that lasted up to 2 min, local sensitivity, and lower abdominal cramping, have been noted following CO_2_ laser therapy [16]. In our trial, women reported higher pain scores and vaginal spotting after laser therapy compared with sham procedures, but these effects were generally mild and short lived. No major adverse effects were observed.

In the current study, all vaginal laser procedures were performed by urogynecologists following a standardized protocol, minimizing variability in treatment quality and technique. This consistency is a strength of our study. Additionally, the multicenter design helps to mitigate selection bias by incorporating diverse geographic locations, facilitating comparisons across different population groups and enhancing the generalizability of our findings. Besides, several confounding factors were addressed in our analysis including the compliance of pelvic floor exercise during the study period. Another notable strength is the implementation of a single-blinded study with sham treatment as a control, which enhances the study's ability to provide high-quality clinical evidence. Besides, we achieved a high level of compliance with our trial protocol, and our loss to follow-up rate was lower than expected, likely because of the geographical advantages of the study center and low consultation costs in public hospitals in our locality.

We acknowledge certain limitations in our study. A confounding factor that has been considered as a predictive factor of treatment success [19], such as weight changes during the study period, was not included and could potentially influence treatment outcomes. To enhance the robustness of the trial, this confounding factor should be considered in future research. Although we disclosed group assignments post-study, we did not evaluate participants'perceptions during the study. This may limit our understanding of blinding efficacy. We recognize that assessing these perceptions could offer valuable insights for future studies. Furthermore, 60% of participants were sexually inactive and did not complete the FSFI questionnaire. As a result, there was a lack of information related to sexual function outcomes.

Vaginal laser therapy is a minimally invasive procedure that can be performed on an outpatient basis, allowing women to avoid hospital stays and resume normal activities within a few days. We propose that vaginal laser therapy could be considered a viable treatment option for women with persistent SUI symptoms who have not responded to pelvic floor exercises or pessaries and may not be suitable candidates for surgical interventions. There is potential for further exploration of the efficacy of Erbium:YAG laser, CO_2_ laser, and vaginal estrogen treatments. Additionally, the cost-effectiveness of vaginal laser therapy needs to be evaluated before it can be widely implemented in clinical practice.

## Conclusions

This multicenter randomized controlled trial found that vaginal Erbium:YAG laser treatment provided significant subjective and objective improvements in women with SUI after 12 months. The therapy was well-tolerated, suggesting its potential as a minimally invasive option. However, the results highlight the need for further research to explore its efficacy in diverse populations and to better understand the influences of patient expectations and the placebo effect on treatment outcomes.

## Data Availability

The trial data are available from the corresponding author upon request to facilitate future investigation.
